# High-Performance and Fabrication-Tolerant 3 dB Adiabatic Coupler Based on Ultralow-Loss Silicon Waveguide by Tri-Layer Hard Mask Etching Process

**DOI:** 10.3390/nano15120947

**Published:** 2025-06-18

**Authors:** Ke Zhang, Yunchu Yu, Nanfei Zhu, Senlin Zhang, Jie Sun, Shijin Ding, David Wei Zhang

**Affiliations:** 1 School of Microelectronics, Fudan University, Shanghai 200433, China; 20112020041@fudan.edu.cn; 2 Silith Technology Co., Ltd., Shanghai 201210, China

**Keywords:** silicon photonics, ultralow loss, waveguide, adiabatic directional coupler

## Abstract

Silicon photonics has emerged as critical for advancing photonic integrated circuits (PICs), but waveguide losses, primarily resulting from sidewall roughness, remain a primary challenge. In this work, we demonstrate a tri-layer hard mask etching process that produces strip silicon waveguides with propagation losses as low as 1.48 dB/cm, i.e., a 37% improvement over the conventional Si_3_N_4_ hard mask technique. Based on the abovementioned approach, the fabricated 3 dB adiabatic directional couplers achieve a nearly ideal splitting ratio of 50.2:49.8 as well as an excess loss of 0.067 dB. These results indicate that the tri-layer hard mask etching process enables scalable and ultralow-loss PICs to be fabricated for high-speed optical interconnects and quantum computing systems.

## 1. Introduction

Silicon-on-insulator (SOI) is a promising platform for large-scale photonic integrated circuits (PICs) due to its strong optical field confinement and complementary metal–oxide–semiconductor (CMOS) compatibility [1,2,3]. As the core component in silicon photonics technology, optical waveguides serve as essential components for efficient optical signal propagation. Reducing optical waveguide loss is regarded as the most critical challenge in the development of PICs, and great efforts have been made to develop ultralow-loss optical waveguides in the past decades [4]. In general, SOI waveguide losses originate from scattering, radiation, and mode-mismatch, corresponding to the roughness of the waveguide sidewalls, waveguide bending, and the mode field difference between the bent and straight waveguides, respectively [5]. Among them, reducing the waveguide sidewall roughness by improving the fabrication process or implementing specialized processes has proven effective in reducing the waveguide losses, such as oxidation-based etch-less processes [6], chemical oxidation [7], anisotropic etching [8], and post-processing such as thermal oxidation and annealing [9,10]. Another strategy is to reduce the interaction of the optical mode field with the rough sidewalls by introducing shallowly etched ridges or ultra-thin core regions [7,11,12].

Power splitters play crucial roles in various applications including optical modulation, wavelength-division multiplexing (WDM), switching, and passive optical networks (PONs) [13,14,15,16]. However, in large-scale optical switches and neural networks, the extensive use of splitters exacerbates insertion loss and crosstalk, thus degrading the system performance [17,18]. Common splitter designs include Y-junctions [19], directional couplers (DCs) [20], multimode interferometers (MMIs) [21], and adiabatic directional couplers (ADCs) [22]. The Y-junctions feature compact dimensions for dense integration, but exhibit mode-mismatch-induced losses unless junction angles are minimized [19]. The DCs use abrupt coupling between adjacent waveguides, achieving low loss and compactness but suffering from wavelength sensitivity and narrow bandwidth [23,24]. The MMIs utilize self-imaging principles for wavelength-insensitive operation and high fabrication tolerance but struggle with asymmetric power splitting [22,25]. While structural optimization enables arbitrary MMI splitting ratios, their insertion losses typically exceed those of the DCs. The ADCs achieve broadband coupling via adiabatic mode evolution but require lengths exceeding 240 µm and exhibit high insertion losses [26,27].

The tri-layer hard mask etching process is a key graphic transfer technique in CMOS technology, mainly used for the etching of high-precision, high-depth-to-width-ratio structures, and is especially widely used in the fabrication of integrated circuits at advanced nodes (e.g., 28 nm and below) [28,29,30]. As far as the current silicon optical technology using backward technology nodes is concerned, the THME process is promising in improving the roughness of the sidewalls of the optical waveguide as well as reducing the loss. Considering the widespread deployment of power splitters in PICs, the minimization of excess loss, footprint, and fabrication complexity becomes paramount. We demonstrate a tri-layer hard mask etching technique using spin-on carbon (SOC), silicon anti-reflective coating (SiARC), and photoresist layers. This approach enables precise sidewall profile control, achieving a line-edge roughness (LER) of 8.51 nm and propagation loss as low as 1.48 dB/cm. The optimized ADCs maintain a splitting ratio of 3 ± 0.15 dB in the wavelength range of 1500–1600 nm together with 0.067 dB excess loss. This work paves the way for scalable fabrication of low-loss photonic devices, advancing energy-efficient optical communications and computing.

## 2. Tri-Layer Hard Mask Etching Process

Fabrication was performed on 200 mm silicon-on-insulator (SOI) wafers from SOITEC(Bernin, France) comprising a 220 nm thick single-crystal silicon device layer atop a 3 µm buried oxide (BOX) layer. Critical dimension and line-edge roughness (LER) measurements used CD-SEM (CG5000, Hitachi High-Tech Corporation, Tokyo, Japan) at 5 kV with 1 nm resolution. Propagation losses were quantified via the cutback method using a tunable laser source (81600B, Keysight Technologies, Santa Rosa, CA, USA), a polarization controller (Keysight N7786), an optical power meter (N7744A, Keysight Technologies, Santa Rosa, CA, USA), and a wafer prober (SUMMIT200, FormFactor, Inc., Livermore, CA, USA). TE-polarized light was injected into the waveguides. We designed two spiral waveguides with different lengths: a short waveguide (*L*_short_ = 4 cm) and a long waveguide (*L*_long_ = 9 cm), with a length difference of 5 cm. The propagation loss α (in dB/cm) is calculated as:

(1) $$\alpha =\left[P_{\mathrm{out}}\left(L_{\mathrm{short}}\right)-P_{\mathrm{out}}\left(L_{\mathrm{short}}\right)\right]/\left(L_{\mathrm{long}}-L_{\mathrm{short}}\right)$$

where *P*_out_(*L*_short_) and *P*_out_(*L*_long_) are the output powers for the short and long waveguides, respectively.

**Figure 1:**
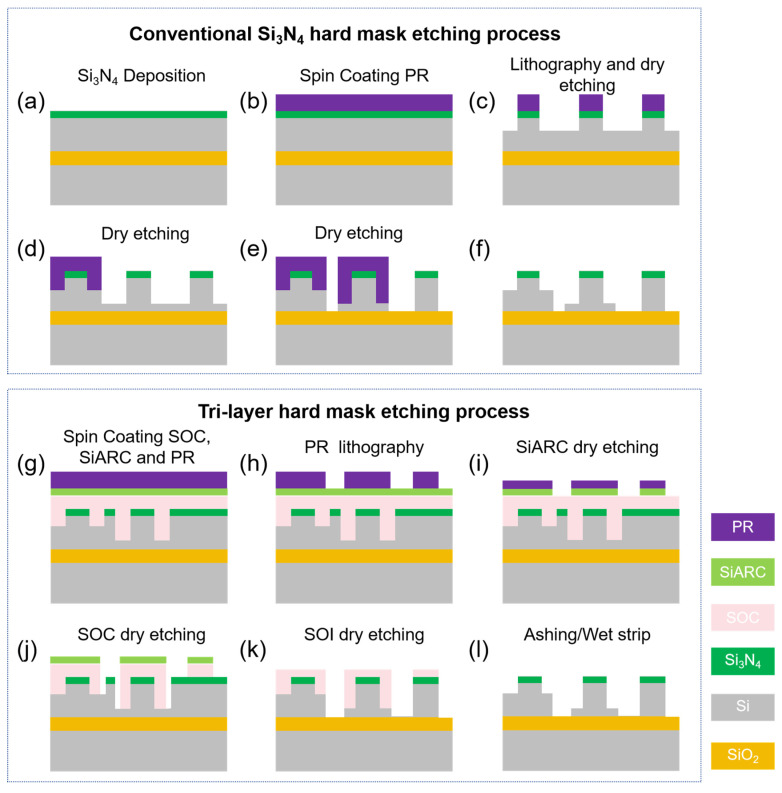
(**a**–**f**) Schematic diagram of conventional Si_3_N_4_ hard mask etching process. (**g**–**l**) Schematic diagram of tri-layer hard mask etching process.

**Figure 2:**
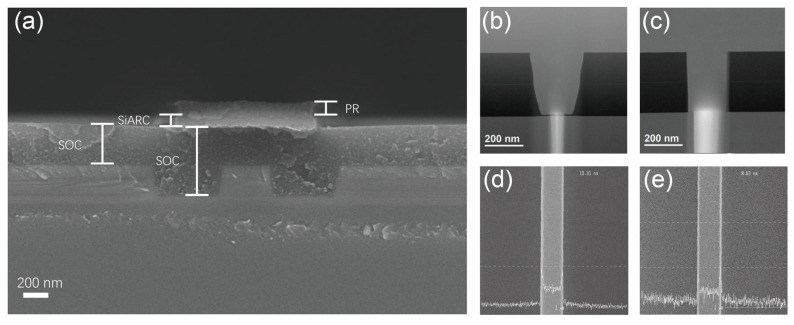
(**a**) Cross-sectional SEM image of a half-etched ridge-type waveguide after etching the SiARC layer in a tri-layer hard mask process flow. (**b**) Cross-sectional SEM photographs of a strip waveguide prepared by a conventional Si_3_N_4_ hard mask process. (**c**) Cross-sectional SEM photographs of strip waveguides prepared by tri-layer hard mask process. (**d**) CDSEM results of a strip waveguide prepared by a conventional Si_3_N_4_ hard mask process. (**e**) CDSEM results of a strip waveguide prepared by tri-layer hard mask process.

Figure 1a–f depict the flow schematics of the conventional Si_3_N_4_ hard mask process, where multiple etching steps typically induce a cumulative increase in the waveguide sidewall roughness, resulting in markedly enhanced optical scattering loss of waveguide propagation. Figure 1g–l illustrates the process flow of the tri-layer hard mask etching process proposed in this work, in which a SOC layer and a SiARC are sequentially deposited on both shallowly and deeply etched ridge waveguides. The cross-sectional SEM image of the SiARC layer after etching in the tri-layer hard mask process flow is shown in Figure 2a, in which the topmost region is photoresist (PR), with a thickness remaining of about 55 ± 3 nm after etching, and the bottom of the PR is 45 ± 2 nm thick SiARC. Since there is still a residue of PR, it effectively defines and preserves the SiARC layer, which is conducive to using SiARC as a mask layer for the subsequent etching process. The thicknesses of the SOC layer in different regions are 323 ± 16 nm (left side) and 540 ± 27 nm (half-etched waveguide region), respectively, and it exhibits thickness variations in different locations. This difference is due to the uneven bottom caused by the half-etched waveguide, and the SOC acts as a hard mask to fill in the bottom area, effectively transferring the pattern and maintaining mechanical stability. Overall, the interface between the photoresist and the SiARC layer, as well as the SOC layer, is clear, and the tri-layer hard mask etching process addresses a critical fabrication challenge, i.e., the flatness of the different waveguide regions.

Figure 2b and Figure 2c present the scanning electron microscopy (SEM) morphologies of the strip waveguides fabricated with the conventional Si_3_N_4_ hard mask and the tri-layer hard mask etching process, respectively. The former results in excessive angulation of the waveguide sidewalls and appears to visibly form depressions, which visually increase the roughness of the sidewalls. This may be due to the difference in the etching rate and selection ratio of the different etching steps, which may result in the formation of bumps or depressions at the junction. The latter results in the strip waveguide with flat and vertical sidewalls, and no significant shrinkage or sharp corners are observed. Figure 2d,e display the sidewall roughness of the 400 nm wide strip waveguide using critical dimension scanning electron microscopy (CDSEM) for both processes. The tri-layer hard mask etching process achieves a significant reduction in line-edge roughness of the strip waveguide compared to the conventional silicon nitride hard mask process, i.e., from 13.48 nm to 8.51 nm.

**Figure 3:**
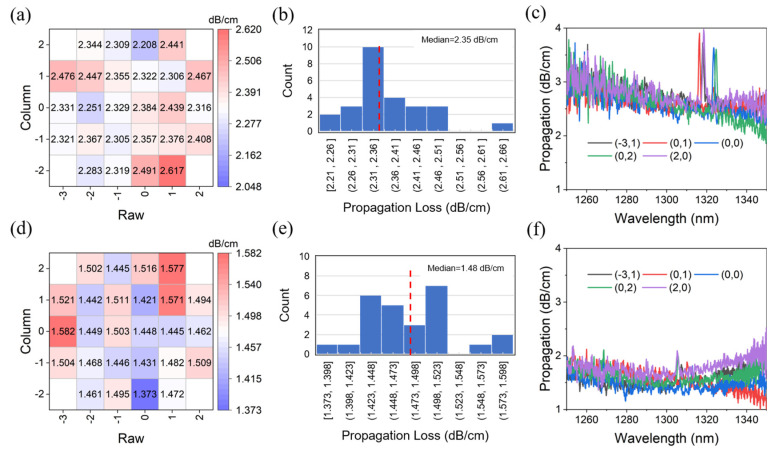
(**a**) Thermal map of waveguide loss distribution at 1311 nm for strip waveguides fabricated using conventional Si_3_N_4_ hard mask process. (**b**) Histogram of waveguide propagation loss under conventional Si_3_N_4_ hard mask process. (**c**) Plot of propagation loss versus wavelength for strip waveguides using conventional Si_3_N_4_ hard mask process. (**d**) Thermal map of waveguide loss distribution at 1311 nm for strip waveguides fabricated using tri-layer hard mask process. (**e**) Histogram of waveguide propagation loss under tri-layer hard mask process. (**f**) Plot of propagation loss versus wavelength for strip waveguides using tri-layer hard mask process. M and S are abbreviations for median and standard deviation, respectively. (x,y) represents the die at the xth row and yth column position on the wafer.

To comprehensively compare the effects of the conventional silicon nitride hard mask and tri-layer hard mask etching processes on waveguide propagation loss, wafer-level testing of the propagation loss of strip waveguides on a 200 mm SOI platform was targeted. The TE polarized light from a tunable laser was injected into waveguides with different lengths and the output was measured by an optical power meter. The famous cut back method was used to extract the unit propagation loss of the waveguide. Figure 3a and Figure 3d show the heat map of the propagation loss distribution at the 1311 nm wavelength on a 200 mm wafer (containing 26 chip regions) before and after waveguide process optimization, respectively. The histograms in Figure 3b,e further validate the median propagation loss before and after waveguide process optimization. The tri-layer hard mask etching process improves the median propagation loss from 2.35 dB/cm to 1.48 dB/cm. The tri-layer hard mask etching process produces lower strip silicon waveguide losses and a more uniform loss distribution. However, there are localized regions of higher loss at the periphery of the wafer, which may be related to the pattern mismatch due to the etching uniformity. In addition, the loss distributions and histograms of deeply etched ridge-type waveguides and shallowly etched ridge-type waveguides on wafers are also investigated, as shown in Figure S1. On the one hand, the waveguide loss under the tri-layer hard mask process is significantly better than that of the Si_3_N_4_ hard mask, with the median losses of 1.18 dB/cm and 0.29 dB/cm for the deep-ridged and shallow-etched ridge waveguides, respectively. On the other hand, the thermal map distribution shows that the deeply etched ridge-type waveguides also suffer from the similar problem of high localized loss, while the shallowly etched ridge-type waveguides do not have such a problem. Therefore, there is still room for optimization of the tri-layer hard mask process in terms of etching quality and mode conversion. In contrast, the uneven loss distribution exhibited by the Si_3_N_4_ hard mask etched strip silicon waveguide should originate from the limitations of the deep etching process, which makes it difficult to achieve consistent process control across the entire wafer range, leading to small etch depth differences in different regions. Figure 3c and Figure 3f show the spectral results of the propagation loss of the strip silicon waveguide fabricated using the two processes, respectively. Compared with the conventional Si_3_N_4_ hard mask process, the tri-layer hard mask etching process achieves lower propagation loss in the wavelength range of 1260–1340 nm, especially in the short wavelength range of 1260–1280 nm. In the wavelength range of 1280–1300 nm, the loss of the strip waveguide is typically higher, but the tri-layer hard mask etching process still achieves a 30–40% reduction compared to the Si_3_N_4_ mask process. Therefore, combined with the optimization results of the tri-layer hard mask Si_3_N_4_ process for line-edge roughness and propagation loss, the process excels in reducing scattering loss, increasing pattern stability, and improving fabrication uniformity, making it particularly suitable for broadband low-loss propagation applications.

## 3. Adiabatic Directional Couplers

According to the results discussed above, the tri-layer hard mask technique can not only significantly reduce the sidewall roughness of silicon waveguides, but also can realize perfectly flat and vertical profiles of the waveguide cross-section. One should expect a certain excess loss reduction since the length of ADC is generally large. In addition, the ideal geometrical profile will suppress the unwanted supermodes coupling as designed, which will ensure a balanced splitting ratio for the fabricated ADC. To validate the effectiveness of tri-layer hard mask technology on the fundamental silicon photonics component, we designed and fabricated adiabatic directional couplers (ADCs) on the 200 mm SOI platform.

**Figure 4:**
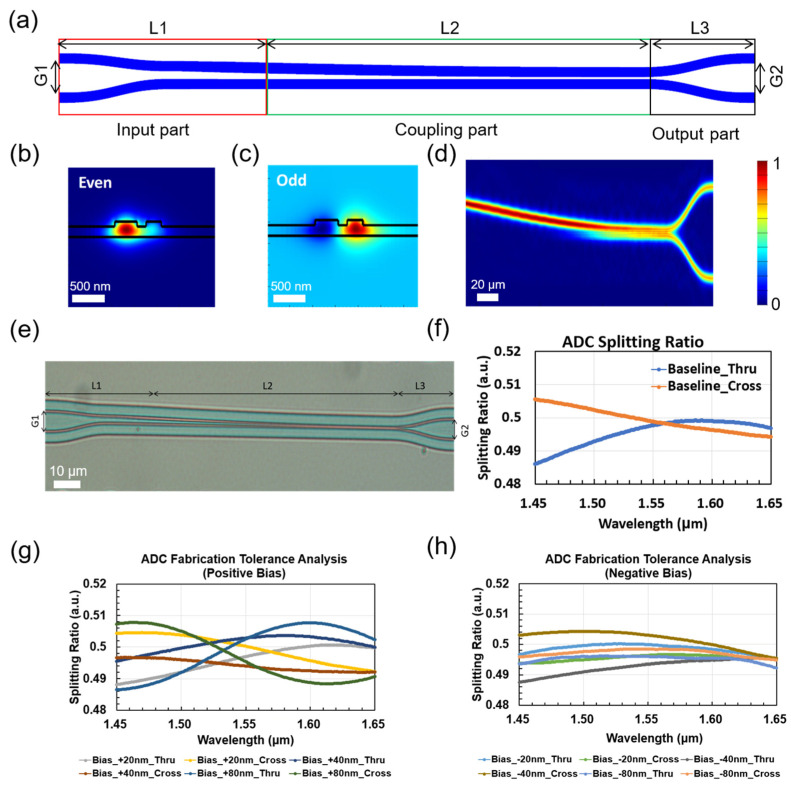
(**a**) Schematic view of ADC coupler design. (**b**–**d**) Simulated electric field distributions for the wavelength of 1550 nm, when the light is launched into Input 1: (**b**) even; (**c**) odd and (**d**) top view. (**e**) Optical micrograph of the ADC. (**f**) Simulated ADC splitting ratio. (**g**,**h**) Simulated splitting ratios versus wavelength for different coupler lengths.

The schematic structure of the proposed low-loss waveguide-based ADC is depicted in Figure 4a. Generally, an ADC is composed of three parts, i.e., an input part, coupling part, and output part. The coupling part contains two waveguides whose widths are slowly changed from the left side to the right side. Such a structure can be treated as a supermode waveguide system. The coupling part should be judiciously designed to suppress the coupling between the supermodes supported by the waveguide system to ensure a low insertion loss and balanced splitting ratio performance. The supermodes coupling intensity can be expressed as follows:

(2) $$\Omega =\underset{m\neq n}{\sum }|\int_{0}^{1}\kappa_{mn}\left(z\right)e^{jL\int_{0}^{L}|\beta_{m}\left(s\right)-\beta_{n}\left(s\right)|ds}dz{|}^{2}$$

where *L* is the coupling length, $\beta_{m}\left(z\right)$ and $\beta_{n}\left(z\right)$ are the complex propagation constant of supermode *m* and *n*, and $\kappa_{mn}$ is the localized coupling coefficient of mode *m* and *n*,

(3) $$\kappa_{mn}\left(z\right)=\frac{\omega }{4}\frac{1}{\beta_{m}\left(z\right)-\beta_{n}\left(z\right)}\int {\bar{E}}_{m}^{*}\;\frac{\partial \varepsilon }{\partial z}{\bar{E}}_{n}dxdy$$

where ω is the angular frequency, ${\bar{E}}_{m}$ and ${\bar{E}}_{n}$ are the electrical field of supermode *m* and *n*, and ε is the complex dielectric constant. To realize a minimum coupling coefficient $\kappa_{mn}$, the difference between $\beta_{m}\left(z\right)$ and $\beta_{n}\left(z\right)$ should be designed as relatively large. Following the general rule discussed above, the optimized parameters of the ADC are as follows: G1 and G2 are 7.4 μm and 7.5 μm, and L1, L2, and L3 are 60 μm, 150 μm, and 40 μm, respectively. The input and output waveguide widths are fixed at 0.5 μm. The upper waveguide tapers from 0.5 μm to 0.8 μm in the input part and then tapers from 0.8 μm to 0.4 μm in the coupling part. The lower waveguide tapers from 0.5 μm to 0.4 μm in the input part and then remains constant in the coupling part. For the output part, both waveguides taper from 0.4 μm to 0.5 μm.

Figure 4b,c depict the electric field distribution of the 1st and 2nd supermodes of the waveguide system. The cross-section is chosen to be the left port of the coupling part. The even mode corresponds to the 1st supermode, and the odd mode corresponds to the 2nd supermode. The electric field of these two modes are quite different as a result of our dedicated design. Figure 4d illustrates the static optical intensity propagation when light is injected from the upper waveguide at the left port of input part of the designed ADC. Apparently, a balanced optical intensity output is observed at the ADC right output ports. Figure 4e shows a micrograph of an ADC based on a low-loss waveguide process. Figure 4f shows the simulated splitting ratio between the two ADC ports. The splitting ratio of both ports of this ADC is within 3 dB ± 0.2 dB in the range of 200 nm from 1450 nm to 1650 nm, and 3 dB ± 0.15 dB in the range of 100 nm from 1500 nm to 1600 nm. Figure 4g,h show the simulation analysis of the processing tolerance of the ADC, demonstrating negligible degradation even with ±80 nm waveguide width variations.

**Figure 5:**
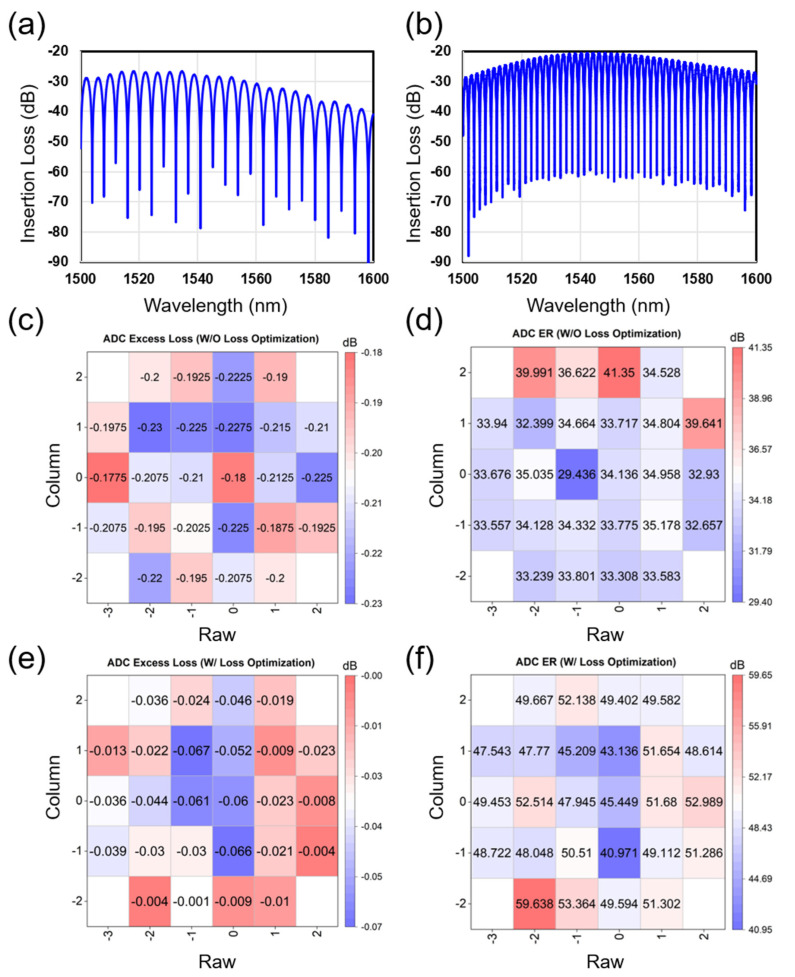
Tested results of ADC without loss optimization: (**a**) optical interference spectrum; (**c**) excess loss wafer map; and (**d**) ER wafer map. Those of ADC with loss optimization: (**b**) optical interference spectrum; (**e**) excess loss wafer map; and (**f**) ER wafer map.

To test the splitting ratio of ADC, a cascaded ADC Mach–Zehnder interference (MZI) structure is designed in this paper. The MZI structure consisted of two cascaded ADCs connected by two arms of different length. The length differences resulted in interference fringes as shown in Figure 5a,b. Since the length differences are 140 μm and 308 μm, so the free spectral range (FSR) of the interference fringes in Figure 5a,b are slightly different. However, one should note that such a difference will not affect the splitting ratio evaluation. By testing the ER of the interference structure, the spectral ratio of the ADC can be deduced. The relationship between the ER and splitting ratio can be derived as follows:

(4) $$20\log_{10}(\frac{\sqrt{a}+\sqrt{b}}{\sqrt{a}-\sqrt{b}})=ER$$

where *a* and *b* are the splitting ratio and *a* + *b* = 1.

Figure 5 compares the performance of ADC with and without waveguide loss optimization. Figure 5a,b show the typical interference optical spectrum of ADC without and with waveguide loss optimization. The ER is improved from 30 dB to 40 dB in the range of 100 nm from 1500 nm to 1600 nm, which means the splitting ratio is improved from 3 dB ± 0.28 dB to 3 dB ± 0.15 dB. Figure 5c,e compare the excess loss wafer map of ADC without and with waveguide loss optimization. The excess loss is improved from -0.23 dB to larger than −0.067 dB. As shown in Figure 5d,f, after waveguide loss optimization, the extinction ratio values range from 40.971 dB to 53.638 dB, and the difference in extinction ratios at each position is reduced.

Table 1 summarizes the performance of state-of-the-art 3 dB adiabatic couplers. While the device size of this work is not minimal, it achieves an excellent beam splitting ratio and sufficiently low additional loss given the smaller size. The advantages of the low-loss waveguide process for the comprehensive performance enhancement of ADC devices are fully demonstrated, which is highly valuable and promising for applications in the fields of large-scale, multistage optical switching arrays and optical neural networks.

## 4. Conclusions

Silicon photonics has long been constrained by waveguide losses dominated by sidewall roughness, limiting the performance and scalability of photonic integrated circuits. In this work, we introduce a tri-layer hard mask etching process that overcomes this limitation by enabling ultralow-loss silicon waveguides and high-performance photonic devices. By integrating SOC, SiARC, and photoresist layers, the tri-layer hard mask etching process achieves precise control over sidewall profiles, reducing line-edge roughness to 8.51 nm and waveguide propagation loss to 1.48 dB/cm. This advancement stems from enhanced etching uniformity, minimized scattering losses, and improved mechanical stability during fabrication. The tri-layer hard mask etching process proves capable of achieving high performance and fabrication tolerance for one elementary silicon photonics component, i.e., ADCs. The fabricated ADCs achieve a near-ideal splitting ratio of 3 dB ± 0.15 dB with only 0.067 dB excess loss. These devices demonstrate exceptional wavelength insensitivity, fabrication tolerance, and compactness, addressing critical limitations of existing splitters in terms of loss, footprint, and operational bandwidth.

## Figures and Tables

**Table 1:** Comparison of the performance of our device with previously reported silicon photonics 2 × 2 3 dB adiabatic couplers. RAC: rapid adiabatic coupler.

Type	Year	Splitting Ratio	Excess Loss (dB)	Device Length (μm)
ADC [31]	2024	0.563:0.437	0.22	79
ADC [32]	2024	0.538:0.462	0.18	108
ADC [26]	2021	0.53:0.47	NR	110
ADC [33]	2018	0.52:0.48	0.3	70
ADC [34]	2019	0.56:0.44	NR	26.3
ADC [35]	2018	0.533:0.467	NR	100
ADC [27]	2013	0.51:0.49	0.3	300
RAC [36]	2018	0.51:0.49	NR	NR
This Work		0.516:0.484	0.067	89

## Data Availability

The data presented in this study are available on request from the corresponding author.

## Supplementary Materials

The following supporting information can be downloaded at: https://www.mdpi.com/article/10.3390/nano15120947/s1. Figure S1: Propagation losses in deeply etched ridge-type and shallowly etched ridge-type waveguides.
