# Supplementary material for: High-Performance and Fabrication-Tolerant 3 dB Adiabatic Coupler Based on Ultralow-Loss Silicon Waveguide by Tri-Layer Hard Mask Etching Process
Source: Nanomaterials (Basel). 2025 Jun 18;15(12):947. doi: 10.3390/nano15120947 (PMC12196242; doi:10.3390/nano15120947)
Supplement: Supplementary file 1 [file nanomaterials-15-00947-s001.zip › nanomaterials-3657552-supplementary.pdf]

Supplementary Information

High Performance and Fabrication Tolerant 3-dB Adiabatic Coupler Based on Ultralow Loss Silicon Waveguide by Tri-layer Hard Mask Etching Process

Ke Zhang, Yunchu Yu, Nanfei Zhu, Senlin Zhang, Jie Sun, Shijin Ding * and David Wei Zhang *

Fig. S1: Propagation losses in deeply etched ridge-type and shallowly etched ridge-type waveguides.

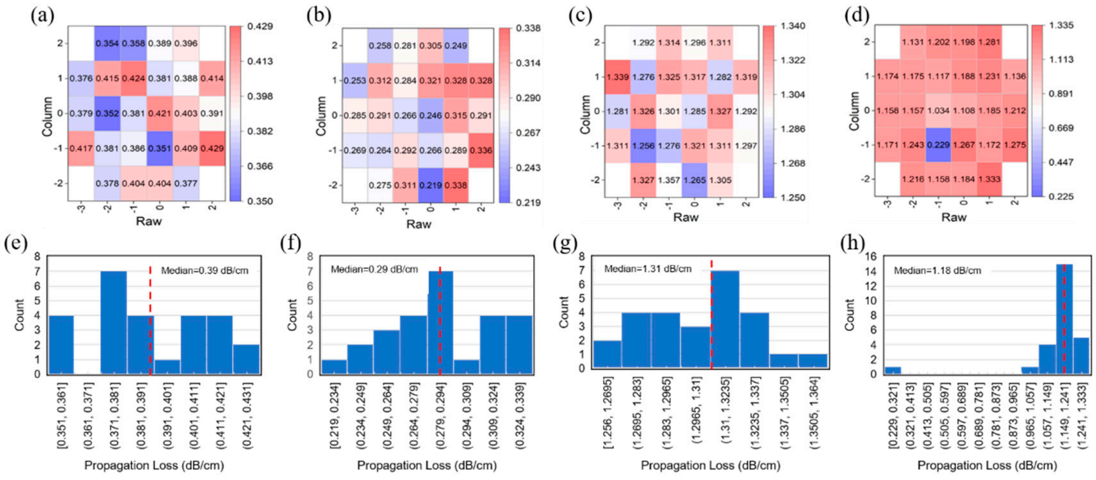

Figure S1. (a), (b) Thermal map of waveguide propagation loss distribution at 1311 nm for shallowly etched ridge-type waveguides fabricated using conventional Si_3N_4 hard mask process and tri-layer hard mask process. (c), (d) Thermal map of waveguide propagation loss distribution at 1311 nm for deeply etched ridge-type waveguides fabricated using conventional Si_3N_4 hard mask process and tri-layer hard mask process. (e), (f) Histogram of shallowly etched ridge-type waveguides propagation loss using conventional Si_3N_4 hard mask process and tri-layer hard mask process. (g), (h) Histogram of deeply etched ridge-type waveguides propagation loss using conventional Si_3N_4 hard mask process and tri-layer hard mask process.
